# Challenges of pre-hospital emergency care at Addis Ababa Fire and Disaster Risk Management Commission, Addis Ababa, Ethiopia: a qualitative study

**DOI:** 10.1186/s12913-024-11292-6

**Published:** 2024-07-11

**Authors:** Feleku Yimer Seid, Birhanu Chekol Gete, Amanuel Sisay Endeshaw

**Affiliations:** 1 Department of Emergency and Critical Care, St. Paul Hospital Millennium Medical College, Addis Ababa, Ethiopia; 2 Department of Anesthesia, College of Medicine and Health Science, Bahir Dar University, Bahir Dar, Ethiopia

**Keywords:** Pre-hospital care, Challenge, EMT, Paramedic, Emergency care, Dispatch center

## Abstract

**Background:**

A challenge to pre-hospital emergency care is any barrier or obstacle that impedes quality pre-hospital care or impacts community pre-hospital utilization. The Addis Ababa Fire and Disaster Risk Management Commission (AAFDRMC) provides pre-hospital emergency services in Addis Ababa, Ethiopia. These services operate under a government-funded organization that delivers free emergency services, including out-of-hospital medical care and transportation to the most appropriate health facility. This study aimed to assess the challenges of pre-hospital emergency care at the Addis Ababa Fire and Disaster Risk Management Commission in Addis Ababa, Ethiopia.

**Methods:**

A qualitative descriptive study was conducted from November 20 to December 4, 2022. Data were collected through in-depth, semi-structured interviews with 21 experienced individuals in the field of pre-hospital emergency care, who were selected using purposeful sampling. A thematic analysis method was used to analyze the data.

**Results:**

This study includes twenty-one participants working at the Addis Ababa Fire and Disaster Risk Management Commission. Three major themes emerged. The themes that arose were the participants’ perspectives on the challenges of pre-hospital emergency care in Addis Ababa, Ethiopia.

**Conclusion and recommendation:**

The Fire and Disaster Risk Management Commission faces numerous challenges in providing quality pre-hospital emergency care in Addis Ababa. Respondents stated that infrastructure, communication, and resources were the main causes of pre-hospital emergency care challenges. There has to be more focus on emergency management in light of infrastructure reform, planning, staff training, and education, recruiting additional professional power, improving communication, and making pre-hospital emergency care an independent organization in the city.

**Supplementary Information:**

The online version contains supplementary material available at 10.1186/s12913-024-11292-6.

## Background

EMS (Emergency Medical Service) systems provide a community’s gateway to acute and emergency medical care for members of the public with time-sensitive conditions, critical illnesses, and injuries. [1] The actions taken to provide medical care are time-dependent, starting with the site of the injury (scene), ambulance transportation, and medical facility treatment. [2]

Pre-hospital emergency medical care has many challenges, including unpredictable patient profiles, emergency conditions, and care administration in a non-medical area. The process before medical interventions, such as justice, stigmatization, dangerous situations, and safe driving; the treatment process, such as triage, refusal of treatment or transport, and informed consent; and the end of life and care are all ethical aspects of pre-hospital emergency medicine challenges. [3–5]

Pre-hospital emergency transportation initiatives and interventions, which include different transportation modes and financial supply, have been implemented in different low and middle-income countries (LMICs) [5, 6] by government and nongovernment or private organizations to improve emergency medical transportation and to tackle treatment delay challenges related to the absence effective mode of transportation in pre-hospital care. [7]

In recent years, low-income countries, including Ethiopia, have been suffering from a double burden of communicable and non-communicable diseases, necessitating emergency care for managing injury and chronic disease complications simultaneously. [8, 9] Reports noted that implementing emergency care systems can reduce mortality and disability-adjusted life years (DALYs) by 45% and 35%, respectively, in LMICs. [10]

Pre-hospital emergency care services in LMICs are hampered by a variety of issues, such as distance, fuel costs, nonfunctioning ambulances, unaffordable ambulances, inaccessible ambulances, poorly managed ambulance systems, poor network or communications, lack of awareness of the programmed, use of traditional modes of transportation, perceived response times, and other misconceptions. [11, 12]

The availability and efficacy of an adequate pre-hospital care system in Ethiopia are minimal. However, timely access to health facilities during emergency cases is a crucial element in emergency conditions, and using an appropriate pre-hospital transportation mode has contributed to the reduction of mortality by preventing a delay in treatment. [13, 14] In Addis Ababa city, organizations such as the Red Cross, Addis Ababa Fire and Disaster Risk Management Commission (AAFDRMC), and other private institutions provide pre-hospital medical services for emergency patients or laboring pregnant women. [15, 16] The AAFDRMC is responsible for providing pre-hospital emergency services in Addis Ababa, Ethiopia. These services operate under a government-funded organization that delivers free emergency services, including out-of-hospital medical care and transportation to the most appropriate health facility. The AAFDRMC has one central dispatch center for fire and pre-hospital services, eight ambulance stations, and about 32 ambulances. The authority provides free pre-hospital care, including scene-to-health facility and inter-facility transfers, with care providers being nurses with short-term pre-hospital patient care training. The AAFDRMC also aims to improve health facilities’ response capacity and preparedness to cope with the challenges at the time of a disaster, such as mass-casualty incidents (MCIs), by implementing emergency and disaster preparedness plans.

In Ethiopia and other Sub-Saharan countries, providing pre-hospital care is beset by various challenges that impede its effectiveness. [17] These include a dearth of Emergency Medical Technicians (EMTs) and certified professional paramedics, inadequately trained ambulance crews, and a suboptimal ambulance system. These challenges further complicate the referral process, leading to patients with emergency cases being transported via ineffective modes of transportation. Those results increase the likelihood of disability, increase the severity of pain, increase the failure rate of rescue, contribute to treatment delay, and result in high morbidity and mortality. [15]

Very few studies have been conducted on pre-hospital emergency care service delivery in Ethiopia. Understanding emergency care service delivery challenges is essential to monitor, evaluate, and implement programs to improve pre-hospital emergency care. This study was intended to explore the challenges of pre-hospital emergency medical care at the AAFDRMC in Addis Ababa, Ethiopia.

## Materials and methods

### Study design, area, and period

An institutional-based qualitative descriptive study was conducted at the Addis Ababa Fire and Disaster Risk Management Commission (AAFDRMC), Addis Ababa, Ethiopia, from November 20 to December 4, 2022.

Addis Ababa is the capital city of Ethiopia. The city has over five million people across eleven sub-cities and over a hundred woredas. The city is located at the heart of the country, at an altitude ranging from 2,100 m at Akaki in the south to 3,000 m at Entoto Hill in the north. Pre-hospital medical service is given by the AAFDRMC, the Ethiopian Red Cross Society, the Addis Ababa branch (ERCS), and a few private, for-profit pre-hospital service-giving organizations, including Tebita Ambulance. The AAFDRMC was established in 1934 G.C. to prevent and control fire and related accidents. In 2008, it started to provide pre-hospital care to the community. It has more than 160 emergency care providers and nine stations, of which the main branch is in the Arada sub-city.

This study is reported per the Consolidated criteria for reporting qualitative research (COREQ) Checklists. [18]

### Participant recruitment

All healthcare professionals, dispatch center workers, ambulance drivers, and case team managers working at AAFDRMC with experience of at least one year were eligible to be included in this study. Emergency healthcare providers who were unavailable during data collection and supportive staff who did not have direct involvement or contact with the emergency care process were excluded.

Participants were recruited using a maximum variation purposive sampling technique, which was used across the different groups of participants to provide a realistic perspective concerning the pre-hospital emergency care challenges. The endpoint for sample selection was information saturation, meaning that further data collection failed to provide additional information or new codes were not developed. The study’s participants were twenty-one AAFDRMC workers, all with at least a year prior experience in pre-hospital emergency care.

### Data collection

#### Development of an interview guide

Data were collected using in-depth, semi-structured interviews with participants regarding the process of EMS in the affected area. An interview guide was prepared after reviewing different literature and reviewed by experts in the field of pre-hospital emergency care. It was initially written in English, then translated into Amharic, and then back to English to ensure consistency. (Supplementary Information 1)

### Training of interviewers

In-depth interviews were conducted by two healthcare providers (emergency nurses) from St. Paul Hospital Millennium Medical College who have experience in pre-hospital medical care for more than a year. A one-day simulation-based training session regarding the interview guide and ethics of qualitative research interviews was given to the data collectors, and the research team closely supervised them.

### Interview process

Interviews with the participants started with their experience regarding pre-hospital emergency care, and according to the interview guidelines, general open-ended questions were asked, for instance, “Describe an infrastructure problem when you participated in pre-hospital emergency response.” Then, depending on the context of the responses, the interviewer continued with probe questions such as “Could you explain more?”

The time and location of the interviews were arranged by agreement with the participants. A total of 21 in-depth interviews were conducted. The interviews varied in length from 31 to 62 min. Interviews were digitally recorded with the informed consent of participants, and the recordings were transcribed verbatim.

Study participants were informed of their right to withdraw from the study at any point. Participants were assigned pseudonym identifiers to ensure anonymity in the research report and any publications. Identifying information was removed from transcripts before analysis.

### Transcription and translation of the data

The data collectors transcribed the audio recordings to the local language, Amharic. To minimize errors and ensure faithfulness to the original recordings, a second data collector reviewed a random selection of transcripts for accuracy To ensure the truthfulness of the transcription process, we presented the transcribed document to the first five participants to check for accuracy. Any discrepancies or missing information identified by the participants were addressed and corrected in the transcripts.

Following participant verification, the Amharic transcripts were translated into English by a professional translator experienced in qualitative research. The translator ensured a conceptually accurate rendering of the data while preserving the original meaning and intent of the participants’ words.

### Data storage

Digital recordings were stored securely on encrypted storage devices. Transcripts were stored electronically on password-protected computers or with access restricted to the research team. Paper copies of transcripts were kept in locked cabinets.

### Data analysis

We applied a thematic analysis method, described by Braun and Clarke, to identify, examine, and report patterns within the data to find themes. [19] Two researchers (FY and BC) performed coding, and the discrepancies raised were handled by discussion. Coding frames were generated after repeated line-by-line reading of the interview transcript. Generated codes and collated them into categories, further refined and organized into potential themes that directly corresponded to the pre-hospital emergency care challenges. Open Code 4.03 software was used in the coding process, enabling the application of clearly defined coding criteria, minimizing ambiguity and enhancing the reliability of the coding scheme.

Trustworthiness was ensured by incorporating various components at each stage of the analysis. Credibility was maintained by faithfully representing the participants’ words and perspectives in the final report. Dependability was established through a detailed description of the research methods, facilitating study replication. Conformability was achieved by assuming a follower role during interviews, allowing participants to shape the discussions, and seeking clarification when needed. Transferability was demonstrated by providing sufficient details about the study site, participants, and data collection methods, allowing readers to assess the potential applicability of the findings to other contexts.

## Results

Twenty-one people were interviewed, of which 67% (14/21) were females, consisting of 13 ambulance nurses, two dispatchers, two ambulance drivers, three case team managers, and one pre-hospital manager. The participants ranged in age from 26 to 59 years, averaging 42.5 (S.D. ±7.60) years. (Table 1).

**Table 1:** Key participant interview profile by department

Sector office	Role in the institution	Frequency *n* (%) *n* = 21	Female *n* (%) *n* = 14
AAFDRMC	Ambulance nurse	13 (61.90)	9 (64.29)
	Ambulance driver	2 (9.52)	1 (7.14)
	Dispatcher	2 (9.52)	3 (21.43)
	Ambulance case team manager	3 (14.29)	1 (7.14)
	Pre-hospital manager	1 (4.76)	0 (0.00)

Pre-hospital emergency medical care challenges were classified under three main themes, including challenges related to infrastructure, communication, and resources for pre-hospital care. (Supplementary Information 2)

### Theme 1: challenges relating to infrastructure

Difficulty to access was because of the geographical condition of the city, the lack of emergency roads in the city, poor road conditions and poor road networks, sharing roads between the public and emergency vehicles, increased travel distance (wide coverage area of branches), a lack of road signs, eroded terrain, and narrow roads. Seasonal difficulties were commonly reported, such as difficulty passing through roads during the rainy season, traffic crowds, and insufficient ambulances at a branch.

*“…Especially around 12 hr. post meridian (pm) local time*,* it is difficult to arrive at the scene due to traffic congestion*,* a lack of emergency roads*,* and narrow roads; as a result*,* the patient receives emergency care for a longer period ”. p01 (Ambulance nurse)*

*“…During the rainy season*,* roads around the city’s outskirts become very muddy or are destroyed by floods*,* making timely access to the scene difficult”. p07 (Ambulance driver)*

*“…Due to the lack of a different route for ambulances and low community awareness regarding the use of an ambulance*,* it is difficult to reach the site quickly when an emergency call comes in.” p17 (Ambulance driver)*

*“…The Kaity Fire and Emergency Rescue Branch has a large catchment area. Referral hospitals are located far from health facilities*,* and the institution has only two ambulances for its large catchment area*,* making it difficult to address many calls in the short time interval available. For example*,* the Trunesh Beijing Hospital is far from the Catholic and Bisrate Gabriel referral catchment areas; these health facilities and emergency patients deteriorate during the journey” p15 (Ambulance case team manager)*

*“…The shortage of trained paramedic professionals and EMT staff*,* as well as the ineffective distribution of emergency pre-hospital centers*,* are major challenges that this center faces and must be addressed as soon as possible.” p20 (Ambulance case team manager)*

Most participants also mentioned infrastructural problems within the Addis Ababa Fire and Risk Management Commission that can affect the proper delivery of pre-hospital care. The problem includes no appropriate ambulance entrance and standing area at local health facilities. Some hospital lifts are crowded and far from ambulance standing areas to transfer patients. Participants reported that the liaison of some hospital areas are hidden and far from the emergency room and labor and delivery rooms, and wheelchair or stretcher roads were poorly constructed.

*“…When we arrive at the hospital*,* there is no ambulance standing area. The corridors are not conducive to riding stretchers… The liaison room is hidden and difficult to find… This causes a delay in handing over the patient on time”. p6 (Ambulance nurse)*

In a fire and emergency rescue commission institution, infrastructure challenges include no appropriate standing place for ambulances, no shower room, no laundry service room, no appropriate pits to store wastes and no incineration places, no properly prepared area for ambulance washing, and no nursing station near the site of an ambulance standing place.

*“… In our branch*,* there is no properly prepared ambulance standing area*,* and the ambulance is exposed to the sun at its standing area throughout the day*,* making the interior of the ambulance very hot. And after we leave the hospitals*,* cleaning the ambulance is difficult because there is no ambulance washing area*,* nurse’s shower room*,* or laundry service”. p13 (Ambulance Nurse)*

### Theme 2: challenges relating to communication

Based on the participants’ views, several challenges were pointed out regarding communication. Because the line is free to call, many people continuously call from the community for joking or fake calls. Misinformative incoming phone calls, such as calling ambulances for non-emergency cases, misinformation about the case, misinformation about the scene, not purely heard calls, and false callings were also common.

*“… Many calls come in*,* especially at night*,* and when I answer them*,* most are dropped*,* or I do not know where they are from… Some are for making fun of and insulting others… Some make false calls*,* making the line busy for true callers”. p10 (Dispatcher)*

Another participant from the dispatch center also stated regarding prank calls below

*“One of the most common challenges is that some people call our emergency phone numbers and report false signs and symptoms*,* wasting the staff’s time and energy.” p12 (Dispatcher)*

At the dispatch center, a communication gap identified was misinformation about the type of case, place of call, and catchment area. Since dispatchers are not health professionals and lack skills, they cannot correctly understand medical terms and not forward detailed information to ambulance nurses. The dispatch center forwarded the message to the ambulance nurse after holding it for many reasons; local health facilities and the community also did not correctly tell the status of the patient and type of case when they were communicating with the dispatch person or ambulance nurse, and large areas were not covered because there was no network (telecommunication problem) and there were limitations in radio communications.

*“…Always*,* there is a conflict between the ambulance nurse and local health facility staff because*,* when they make the call*,* they do not forward the type of case and status of the patient correctly to the dispatch centers*,* and vital signs are not correctly recorded”. p5 (Ambulance nurse)*

*“Most of the time*,* the dispatcher has incorrect information about the citation and type of call*,* and they transfer the call to the ambulance nurse very late… As a result of not being psychologically and materially prepared to handle emergency situations correctly”. p14 (Ambulance nurse)*

Shortage of communication technology equipment was also one of the major issues within the institution. There are no GPS and GIS technologies in dispatch or ambulance to identify where the call came from and where the ambulance was reported in adequate communication radios, which are very old and outdated.

*“…We frequently become perplexed when we are dispatched on a mission since Addis Ababa lacks a reliable GPS system. We are unsure of the best and closest route to take to reach the victims”. p03 (Ambulance case team manager)*

*“…We only have one communication radio on each of our two operational ambulances. If a call comes from anywhere and one ambulance goes*,* the left ambulance cannot hear any call or communication from the dispatch center. At this time*,* we use our phone*,* which is very late for communication”. p8 (Ambulance nurse)*

Regarding inter-liaison communication, there was poor or no clear communication between liaisons at health facilities about the case and status of the patient prior to the call to dispatch centers.

*“…When we arrived at the hospital*,* there was little or no communication between the referring health facility and the catchment hospital… Emergency department staff are not voluntary in handing over patients*,* and we make calls to the command post… This prolongs the patient’s access to care”. p09 (Ambulance Nurse)*

### Theme 3: challenges relating to resources

Lack of medical commodities like a shortage of ambulances, modernized ambulances, insufficient portable oxygen, defective vital sign materials, insufficient emergency equipment, and, in some cases, a lack of emergency medication (refill problem) are frequently cited challenges.

*“…When receiving emergency patients from a health facility*,* monitoring their vital signs is extremely difficult due to nonfunctional or a lack of vital sign monitoring materials. And no adequate emergency medication in the ambulance…” p04 (Ambulance nurse)*

Regarding infection prevention, medical materials challenges include the lack of adequate personal protective equipment (PPE), such as gowns and inappropriate gown colors that do not show contamination. Whole covers, face shields, head covers, gloves, boots, eye goggles, and aprons were reported.

*“…You do not receive fluid-proof PPE*,* such as eye goggles. Even if we are handling trauma cases and patients with highly contagious diseases… The majority of what is available to you are cloth uniforms…” p11 (Ambulance nurse)*

Inadequate funding for personal protective equipment in pre-hospital emergency rooms has become a major issue.

*“There is insufficient personal protective equipment due to insufficient funds allocated to this center. Given that all EMS personnel are at the forefront of the fight against the most infectious diseases such as tuberculosis*,* HIV/AIDS*,* and COVID-19*,* and are in direct contact with patients*,* we should not be subjected to such stresses and challenges.” p21 (Ambulance nurse)*

In respect to sanitization and hygiene materials, inadequate sanitizer, alcohol, and bleach were reported.

*“…In pre-hospital emergencies*,* since cross-contamination is very high in the ambulance system*,* infection prevention activity is mandatory*,* but we do not get any alcohol for hand rubs. There is no place prepared to clean an ambulance and stretcher following a trauma arrival…” p02 (Ambulance nurse).*

From the point of view of our participant, financial constraints were the most common problem which can hinder the service. There are not enough year budgets, the pre-hospital does not have its own budget, and any material for service is decided through a process or discussion that takes a long time. Sometimes the commission’s top executives ignore the requested budget or provide a very small budget for purchasing materials.

*“…There is no independent budget for pre-hospital care… To buy emergency medication and materials*,* after many challenges and a long time*,* you get little response or no response at all since they ignore the pre-hospital service… and this makes it difficult to give quality pre-hospital emergency care…” p19 (Pre-hospital care manager).*

According to the participants, human resource/staff-related challenges most commonly highlighted were only a few diploma/degree nurses, no physicians, no paramedic professionals, no training programs to keep educational levels up to date, no training center, no detailed training if new epidemics emerge, no library to read, no internet access, no procedure room to improve skills, no research center in the institution, lack of career development, and no risk payment.

*“…I work as an ambulance nurse. I have six years of experience as a diploma nurse… there is the possibility of further education…The top managers decided that a diploma or level 4 nurse is enough to give pre-hospital care… no B.Sc. nurse*,* no trained paramedics at all… Despite the fact that there is an emergency*,* an epidemic case*,* and no training…” p18 (Ambulance nurse).*

We work in a shifting program with no duty Monday through Friday. “*It is known that working in pre-hospital emergencies has a higher risk of infection and life-threatening injuries… However*,* there is still a payment risk…” p16 (Ambulance nurse)*.

The adverse chain of management includes a lack of collaboration and participation with other organizations; every activity in pre-hospital emergency medical care is only controlled by the top managers of the commissioner, who are nonprofessionals, and middle and bottom managers have no independent decision-making activity.

*“… Pre-hospital care is dependent on the commission*,* and this makes the pre-hospital service poorly developed and addresses community emergency calls easily” p19 (Pre-hospital care manager)*.

## Discussion

The capacity of emergency care services is typically insufficient during severe emergencies and disasters, and local community resources are likely to be overwhelmed. [20] Emergency care may not be available, especially in the early hours following an emergency. [21, 22] In this case, the public’s involvement in delivering first aid may be crucial. In response, some organizations around the world have recently offered civilian-based pre-hospital guidelines, public education, and exercises for communities. [23] Based on participants’ experiences, the results of this study showed that infrastructure plays a vital role in hospital and pre-hospital emergency medical services. In Addis Abeba City, infrastructure issues such as poor road construction and networks, geographic problems, a lack of emergency roads, seasonal problems, traffic jams, and narrow roads are frequently blamed for causing delays or making access to accident scenes or hospitals difficult. Other infrastructure issues with local health facility distance include inadequate ambulance parking, lift issues, and a lack of a liaison room. These factors have also been reported in previous studies in Iran. [2, 24]

Focusing on the challenges relating to communication, an inappropriate telecommunication system, a communication gap or miscommunication, false calling, inadequate communication radios, being nonprofessional dispatchers, a lack of Geographic Information Systems (GIS) and Global Positioning Systems (GPS), and poor communication between and within facilities are important factors that affect it. Various studies have recommended several enhancements to improve communication and situational awareness in emergency response systems. These include modernizing infrastructures, implementing clear and standardized communication protocols, providing training programs for dispatchers, and incorporating GIS and (GPS). [24–28]

Regarding the challenges relating to resources for pre-hospital care, insufficient professional staffing, not enough ambulances and EMS centers, non-paramedic activities and a lack of motivation among some EMS personnel, a lack of resources at EMS dispatch centers, non-health professionals at the dispatch center, and insufficient nurses for substitution are essential barriers. This finding is consistent with the findings of another study. [24, 25, 29, 30] Related studies have recognized the significant stress experienced by paramedics and EMTs, resulting in a lack of motivation to perform their duties effectively. To combat this issue, it has been suggested that investing in staff well-being through competitive salaries, providing opportunities for career development, and fostering a supportive work environment can help alleviate these challenges. [31, 32]

The participants in this study indicated that pre-hospital emergency care was a neglected part of service in the Addis Ababa Fire and Emergency Rescue Commission. The organization’s structural management is entirely non-health professional, resulting in nonprofessional decisions that result in service flaws and poor response. This result is consistent with the study conducted in Gabon [3]. Establishing advisory boards with a strong representation of clinical professionals like paramedics, emergency physicians, and nurses, creating joint committees, improving communication channels, and considering qualified paramedics or emergency medical professionals in leadership positions is paramount to addressing the abovementioned challenges. [33, 34] The participant also demonstrated challenge-related education, such as a lack of training and no opportunity for advancement, resulting in poor staff knowledge. According to the study participants, most Addis Ababa EMS providers have inadequate skills in handling new epidemic emergency cases. Past studies supported this result. [24, 25, 35] Emergency care cross-contamination in the ambulance system is very high, and infection prevention activity is mandatory. From many participants’ points of view, infection prevention and control factors, including lack of PPE, a uniform for a new employee, a shower room, an ambulance washing place, and adequate solutions and detergents, are the main challenges to preventing infection, which was supported by a study conducted in Gabon and Jimma. [3, 35]

To the best of our knowledge, this is a novel study exploring the challenges of pre-hospital emergency medical care in Addis Ababa, Ethiopia, which will significantly impact plans to improve the quality of pre-hospital care. As a limitation of this study due to financial constraints, we did not conduct a focused group discussion, which might have provided a better understanding of pre-hospital medical care challenges.

### Conclusion and recommendations

The findings of this study revealed that the pre-hospital emergency care provided by the Addis Ababa Fire and Disaster Risk Management Commission in previous emergencies was chaotic. It faces several challenges that limit its ability to provide quality emergency care, including traffic congestion, a lack of an emergency road, a significant communication gap, a lack of professional power, and a lack of medical materials.

The establishment of a formal pre-hospital care system and the establishment of pre-hospital emergency care as an independent organization in the city, investment in infrastructure and infrastructure reform, staff training and education, recruiting additional professional power, public awareness campaigns, and more widely available emergency medical training are all viable solutions to the current barriers to access. Further studies should also be conducted using a multi-center approach that includes the views of different stakeholder groups.

### Electronic supplementary material

Below is the link to the electronic supplementary material.

Supplementary Material 1: Interview guide

Supplementary Material 2: Coding tree for thematic analysis

## Data Availability

Data and materials will be shared upon reasonable request.

## Abbreviations

AAFDRMC: Addis Ababa Fire and Disaster Risk Management Commission
EMS: Emergency Medical Services
EMT: Emergency Medical Technician
LMIC: Low- and Middle-Income Countries
NGO: Non-Governmental Organization
