# Supplementary material for: Challenges of pre-hospital emergency care at Addis Ababa Fire and Disaster Risk Management Commission, Addis Ababa, Ethiopia: a qualitative study
Source: BMC Health Serv Res. 2024 Jul 11;24:803. doi: 10.1186/s12913-024-11292-6 (PMC11241940; doi:10.1186/s12913-024-11292-6)
Supplement: Supplementary file 1 — Supplementary Material 1: Interview guide [file 12913_2024_11292_MOESM1_ESM.docx]

**Interview guide (English version)**

**Introduction**

Briefly introduce yourself and the purpose of the interview.

Explain the study's objectives and the role of the interviewee in the study.

Assure the interviewee of confidentiality and anonymity.

**Background**

Can you describe your role in the Addis Ababa Fire and Disaster Risk Management Commission?

How long have you been working in the pre-hospital emergency care sector?

What are the common types of emergencies you encounter in your work?

**Challenges in Pre-Hospital Emergency Care**

What are the main challenges you face in providing pre-hospital emergency care in Addis Ababa?

How do these challenges impact the quality of care provided to patients?

Can you provide specific examples of situations where these challenges have led to negative outcomes for patients?

**Equipment and Resources**

What are the main challenges related to the availability and accessibility of equipment and resources in pre-hospital emergency care?

How do these challenges impact the quality of care provided to patients?

What are some innovative solutions or strategies that could be implemented to address these challenges?

**Coordination**

How well do different stakeholders in the pre-hospital emergency care system collaborate and coordinate their efforts?

What are the challenges faced by dispatch centers in prehospital care, based on your experience and understanding?

Are there any areas where improved collaboration and coordination could lead to better patient outcomes?

What are some potential barriers to effective collaboration and coordination, and how can they be overcome?
