# Supplementary figures and images for: Challenges of pre-hospital emergency care at Addis Ababa Fire and Disaster Risk Management Commission, Addis Ababa, Ethiopia: a qualitative study
Source: BMC Health Serv Res. 2024 Jul 11;24:803. doi: 10.1186/s12913-024-11292-6 (PMC11241940; doi:10.1186/s12913-024-11292-6)

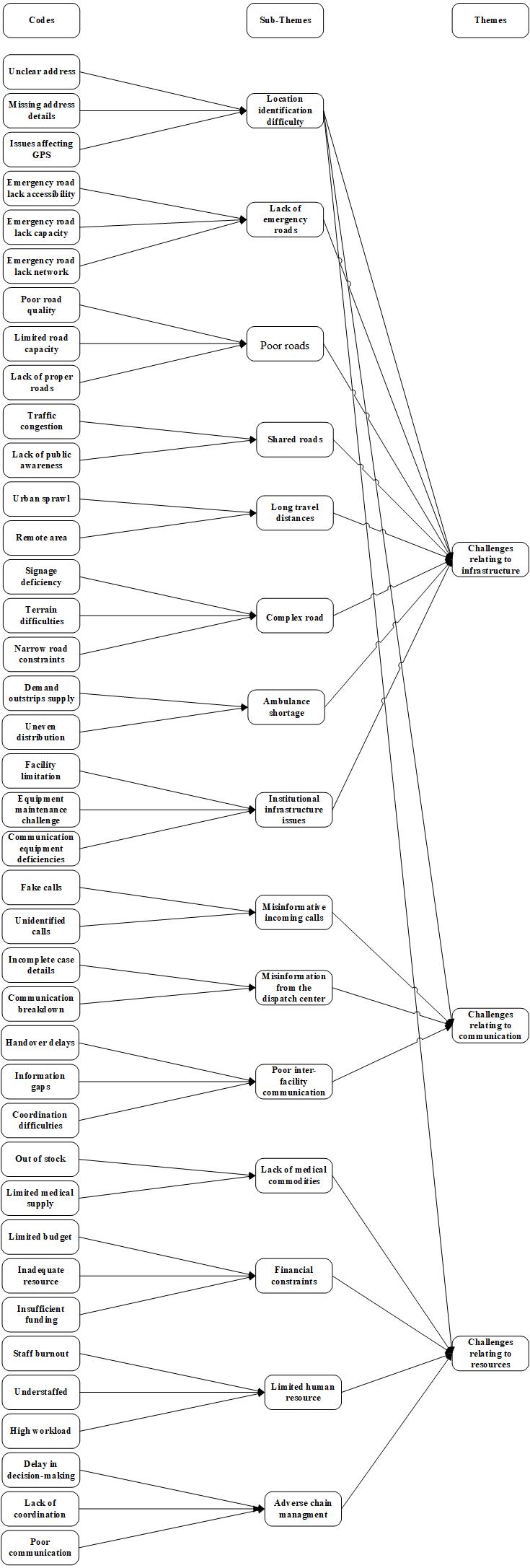

Supplement: Supplementary file 2 — Supplementary Material 2: Coding tree for thematic analysis [file 12913_2024_11292_MOESM2_ESM.jpg]
